# Vascular Leak and Hypercytokinemia Associated with Severe Fever with Thrombocytopenia Syndrome Virus Infection in Mice

**DOI:** 10.3390/pathogens8040158

**Published:** 2019-09-21

**Authors:** Jonna B. Westover, Brady T. Hickerson, Arnaud J. Van Wettere, Brett L. Hurst, Jacqueline P. Kurz, Ashley Dagley, Petra Wülfroth, Takashi Komeno, Yousuke Furuta, Thomas Steiner, Brian B. Gowen

**Affiliations:** 1 Department of Animal, Dairy, and Veterinary Sciences, Utah State University, Logan, UT 84322, USA; jonna.westover@usu.edu (J.B.W.); brady.hickerson@aggiemail.usu.edu (B.T.H.); arnaud.vanwettere@usu.edu (A.J.V.W.); brett.hurst@usu.edu (B.L.H.); jacqueline.larose@usu.edu (J.P.K.); ashley.dagley@usu.edu (A.D.); 2 Utah Veterinary Diagnostic Laboratory, Utah State University, Logan, UT 84341, USA; 3 MChE-Trading Handels-GmbH, Vienna 1060, Austria; petra.wuelfroth@accella-advisors.com (P.W.); thomas.steiner@mche.at (T.S.); 4 FUJIFILM Toyama Chemical Co., Ltd., Toyama 930-8508, Japan; TAKASHI_KOMENO@toyama-chemical.co.jp (T.K.); Yousuke_Furuta@toyama-chemical.co.jp (Y.F.)

**Keywords:** severe fever with thrombocytopenia syndrome, Huaiyangshan banyangvirus, viral hemorrhagic fever, vascular leak, cytokine storm

## Abstract

Severe fever with thrombocytopenia syndrome (SFTS) is an emerging viral hemorrhagic fever (VHF) endemic to China, South Korea, Japan, and Vietnam. Here we characterize the pathogenesis and natural history of disease in IFNAR^-/-^ mice challenged with the HB29 strain of SFTS virus (SFTSV) and demonstrate hallmark features of VHF such as vascular leak and high concentrations of proinflammatory cytokines in blood and tissues. Treatment with FX06, a natural plasmin digest product of fibrin in clinical development as a treatment for vascular leak, reduced vascular permeability associated with SFTSV infection but did not significantly improve survival outcome. Further studies are needed to assess the role of vascular compromise in the SFTS disease process modeled in IFNAR^-/-^ mice.

## 1. Introduction

Severe fever with thrombocytopenia syndrome (SFTS) is an emerging viral hemorrhagic fever (VHF). It is characterized by acute fever, leukopenia and thrombocytopenia with case fatality rates as high as 30% [1,2,3]. The causative agent, SFTS virus (SFTSV), renamed Huaiyangshan banyangvirus of the order *Bunyavirales* (family *Phenuiviridae*, genus *Banyangvirus*), is transmitted by ticks and is closely related to Heartland virus (HRTV), which is endemic in eastern regions of the United States [4,5]. First discovered in China in 2009, SFTSV has also emerged in South Korea, Japan and Vietnam [6,7,8,9]. There are no licensed vaccines or antiviral compounds approved to prevent or treat SFTSV infection.

Common clinical features of SFTS and other VHFs is a prolonged and elevated profile of proinflammatory cytokines in the blood (“cytokine storm”), vascular hyperpermeability, and endothelial barrier dysfunction which can lead to subsequent pathophysiological effects such as disseminated intravascular coagulation and multi-organ failure [7]. Clinical investigation and the study of SFTSV infection of vascular endothelial cells suggest that heightened fluid loss from the vasculature may be due to increased and prolonged gap formation between vascular endothelial cells [10].

To date there are three lethal animal models of SFTSV infection. The immunocompetent age-dependent ferret model mimics many of the clinical manifestations observed in human SFTS cases [11], but limitations of high experiment costs, low availability of aged animals, and facility and caging capacity severely restrict work. A *STAT2* knockout hamster model has been described [12]; however, the limited hamster research reagents constrain the characterization of the host response to infection. An interferon alpha/beta receptor knockout (IFNAR^-/-^) mouse model for SFTSV infection has been reported [13], but clinical features of human SFTS such as vascular hyperpermeability and the proinflammatory cytokine responses were not investigated.

In the present study, we characterized vascular leak associated with infection by the HB29 strain of SFTSV in IFNAR^-/-^ mice and highlight the apparent higher virulence of this strain of virus compared to others that have been studied. In addition, we assessed the cytokine response and show that peak fluid loss from the vasculature correlates with an increase in proinflammatory cytokines. We also evaluated a drug that decreases vascular permeability, FX06, as a monotherapy and in combination with the direct-acting antiviral favipiravir, in an effort to reduce fluid loss from the vasculature and improve survival outcome in mice challenged with SFTSV.

## 2. Results

### 2.1. Lethality of SFTSV Strain HB29 in IFNAR^-/-^ Mice 

**Figure 1:**
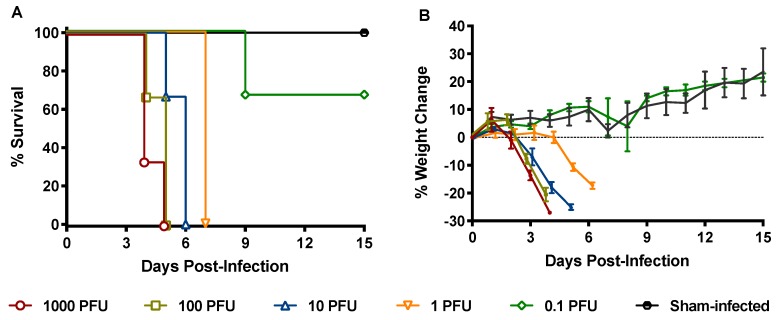
The (**A**) survival outcome and (**B**) percent weight change of IFNAR^-/-^ mice challenged subcutaneously (SC) with SFTSV. Male and female 4-week-old mice (n = 3/group) were challenged SC with the indicated inoculum of SFTSV. Weight change is represented as the group mean and standard error of the percent change in weight of surviving animals relative to their starting weights on day 0, the day of viral challenge.

To determine the 90% lethal dose (LD_90_) of our HB29 virus stock, a small-scale titration of SFTSV in IFNAR^-/-^ mice was performed to guide the design of the full-scale pathogenesis and pathophysiology study to assess vascular permeability, cytokine responses, viral replication and dissemination, and histopathology during the course of infection. Groups of mice (n = 3) were inoculated subcutaneously (SC) with serial dilutions of SFTSV and observed for signs of illness and mortality for 21 days (Figure 1; data shown through day 15). Based on these results, the LD_90_ of the SFTSV HB29 strain in IFNAR^-/-^ mice by the SC challenge route was determined to be approximately 1 plaque forming unit (PFU). Substantial weight loss began 2–3 days post-infection (p.i.) in the groups challenged with 10 PFU or higher and was delayed until day 5 p.i. in the group challenged with 1 PFU of SFTSV (Figure 1B). The delay in clinical disease in the 1 PFU challenge group, as measured by weight loss, was considered for subsequent efficacy experiments on the basis that lower challenge doses would extend the therapeutic window for the evaluation of experimental treatments.

### 2.2. Vascular Leak, Cytokine Response, Virology and Histopathology in IFNAR^-/-^ Mice Infected with SFTSV 

**Figure 2:**
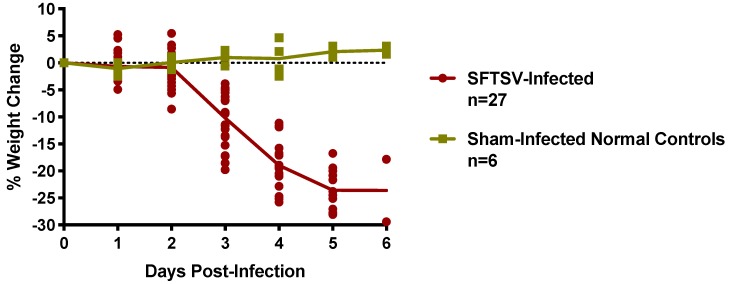
Percent weight change of IFNAR^-/-^ mice challenged SC with 3 PFU of SFTSV. Animals were weighed prior to challenge and randomly assigned to groups for sacrifice on days 2–6. The data are represented as the percent change in weight and group mean of surviving animals relative to their starting weights on the day of challenge. Sham-infected normal controls are included for comparison.

To better characterize the SFTSV infection modeled in IFNAR^-/-^ mice, vascular leak, cytokine profiles, viral titers, and histopathology were analyzed in cohorts of animals challenged with 3 PFU of SFTSV, 3× the calculated LD_90_, to ensure more consistent and uniform disease progression. Challenge groups of three to eight mice each were sacrificed on days 2–6 p.i. to complete the temporal analysis (Supplementary Table S1). Body weights were recorded daily until sacrifice and are reported as the percent change in group mean weights relative to their starting weights on the day of challenge. A notable weight loss of 10% was observed beginning on day 3 p.i. with SFTSV and weight loss approaching 30% was seen on day 6 p.i. (Figure 2).

**Figure 3:**
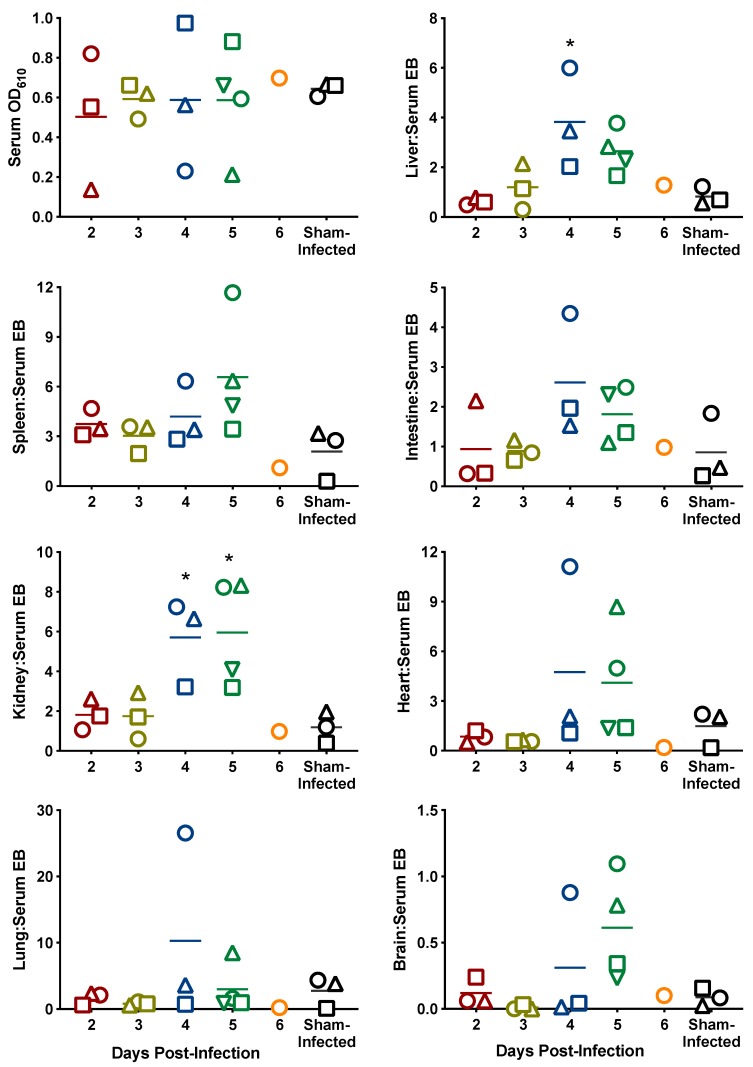
Evaluation of vascular permeability during SFTSV infection in IFNAR^-/-^ mice. Serum data are reported as absorbance at 610 nm minus absorbance at 740 nm. Tissue data are reported as the mean tissue to serum ratio of absorbance/g tissue relative to serum absorbance values. Unique symbols at each sacrifice day represent values for the same animal across serum and all tissues. Serum could not be obtained from three animals in the day-6 sacrifice group that succumbed to the infection prior to sacrifice. * *P* < 0.05 compared to the sham-infected normal controls.

At the time of sacrifice on days 2–6, serum was collected from three to four animals designated for analysis of vascular permeability. As shown in Figure 3, the mean tissue to serum ratio of Evans blue dye (EBD) increased each day in the infected animals, reaching significance in the liver and kidney on day 4. By day 6 p.i. three of the four animals designated for sacrifice that day had succumbed to the infection and the fourth mouse was found in a moribund state. That animal, however, did not have evidence of increased vascular permeability represented by leakage of EBD into the viscera (Figure 3), likely due to the severe dehydration during the terminal stages of disease.

**Figure 4:**
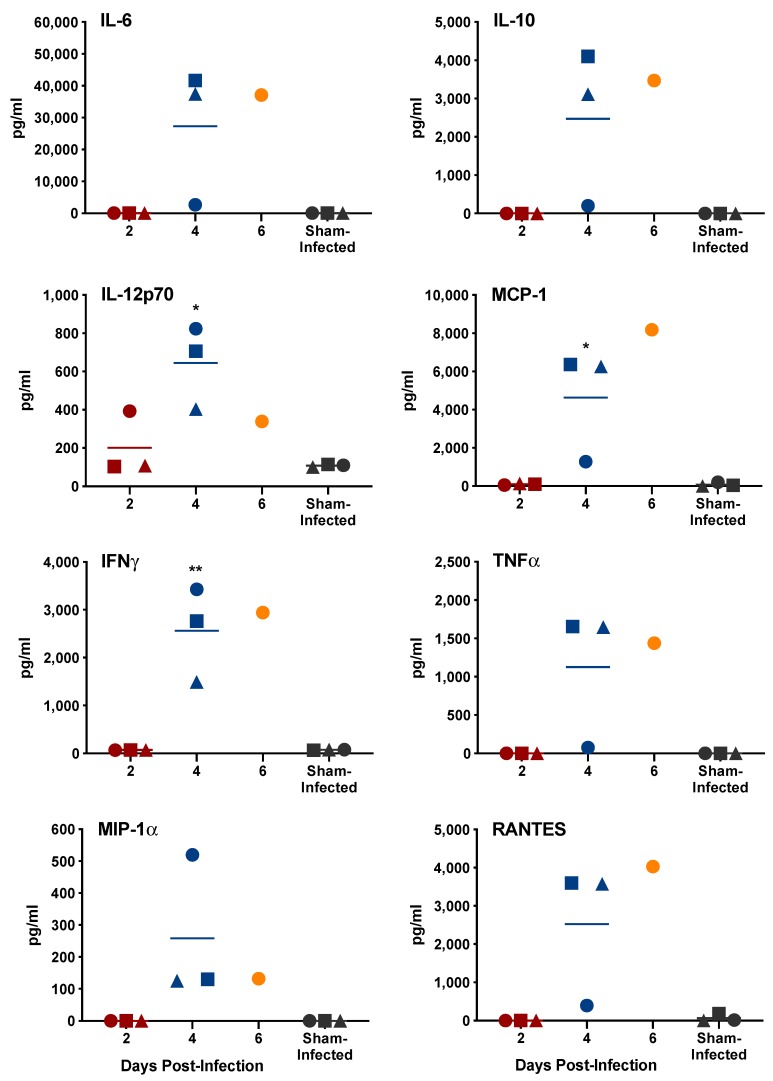
Serum cytokine response to SFTSV infection in IFNAR^-/-^ mice. Unique symbols at each sacrifice day represent values for the same animals across all cytokines. Serum could not be obtained from three animals in the day-6 sacrifice group that succumbed to the infection prior to sacrifice. ** *P* < 0.01, * *P* < 0.05 compared to sham-infected normal controls.

**Figure 5:**
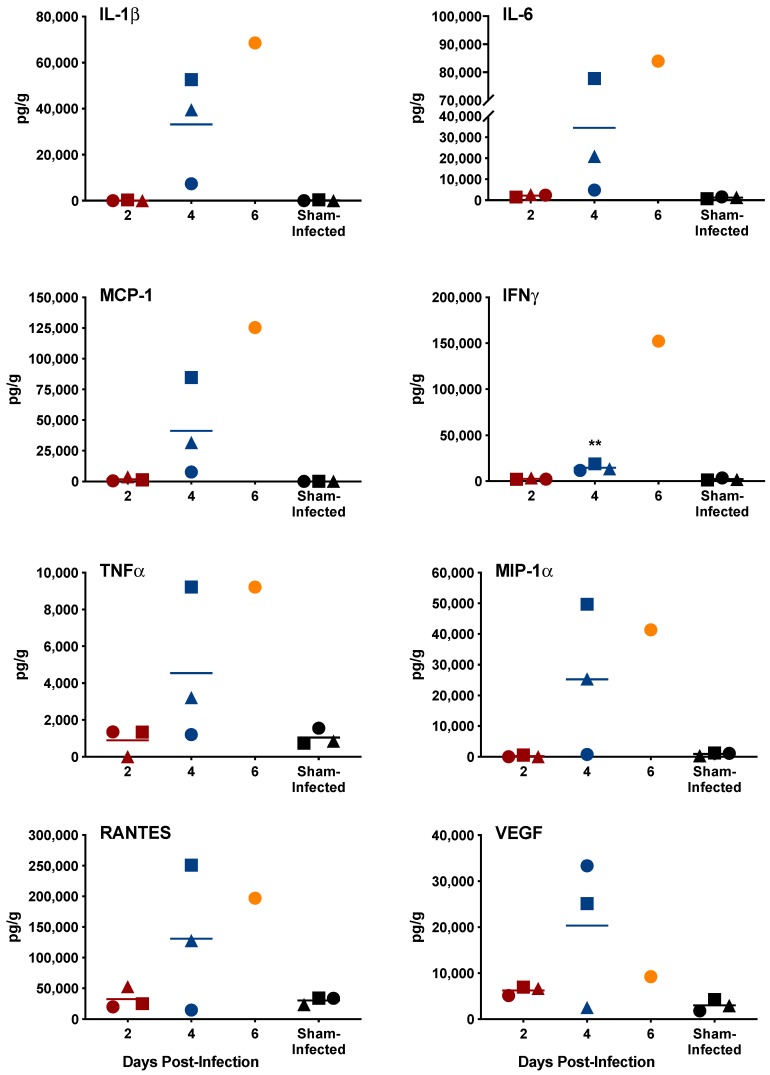
Cytokine response in the spleen to SFTSV infection in IFNAR^-/-^ mice. Unique symbols at each sacrifice day represent values for the same animals across all cytokines. Spleen tissue could not be obtained from three animals in the day-6 sacrifice group that succumbed to the infection prior to sacrifice. ** *P* < 0.01 compared to sham-infected normal controls.

**Figure 6:**
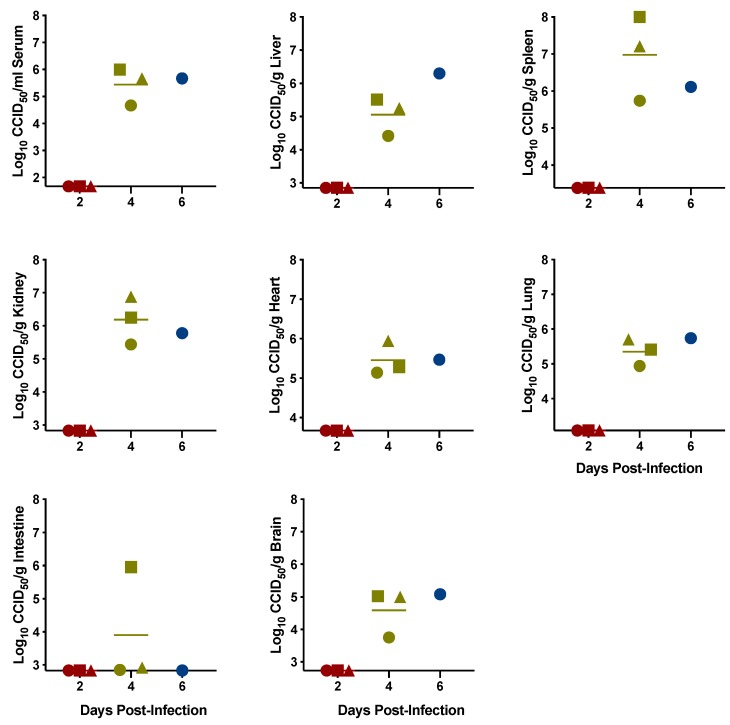
Serum and tissue virus titers in IFNAR^-/-^ mice challenged with SFTSV. Groups of animals were sacrificed on the specified days for analysis of serum, liver, spleen, kidney, heart, lung, intestine, and brain virus titers. The x-axes represent the respective lower limits of detection for serum and the indicated tissues. Unique symbols at each sacrifice day correspond to the same animals across graphs and evaluated parameters in Figure 4 and Figure 5. CCID_50_, 50% cell culture infectious dose.

**Figure 7:**
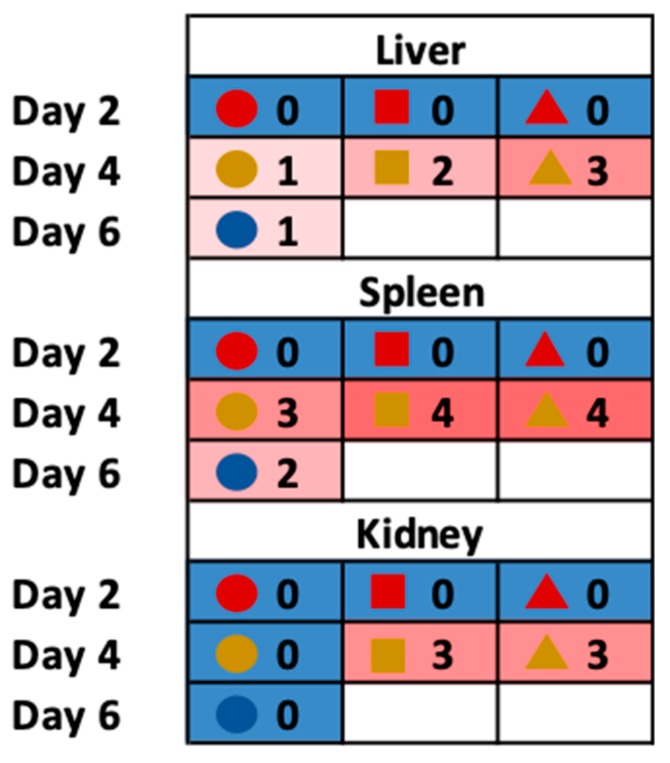
Histopathologic findings observed during the course of SFTSV infection in IFNAR^-/-^ mice. Lesion severity were scored as 0 = no lesions, 1 = minimal, 2 = mild, 3 = moderate, and 4 = severe. No lesions were found in the intestine, heart, lung, or brain (not shown). Hepatic lesions: Acute, multifocal to coalescing, neutrophilic and histiocytic, necrotizing hepatitis. Splenic lesions: Acute, multifocal to coalescing, suppurative and histiocytic splenitis with variable lympholysis, lymphoid depletion, and vascular fibrinoid necrosis. Renal lesions: Acute, multifocal to coalescing tubular epithelial necrosis at the corticomedullary junction and deep cortex. Three animals designated for sacrifice on day 6 succumbed to SFTSV infection prior to the designated time of sacrifice and therefore not included in the analysis. The unique symbols correspond to the same animals across all parameters shown in Figure 4, Figure 5 and Figure 6.

Serum and select tissue cytokine concentrations were examined on days 2, 4 and 6 p.i. in three to four animals per day. Similar to the cohort of animals designated for evaluation of vascular leak, three out of the four animals designated for cytokine profiling on day 6 p.i. succumbed to infection prior to sample collection, limiting our analysis to a single animal at that later time point. A number of prominent proinflammatory cytokines known to be induced during VHF virus infections [14] were found to be increased in the serum on days 4 and 6 p.i. (Figure 4). Many of these proinflammatory mediators, including IL-6, MCP-1, TNFα, IFNγ, RANTES and IL-1β, were also present at high concentrations on days 4 and 6 of infection in the spleen (Figure 5, Supplementary Figure S1), a major target tissue for SFTSV replication (Figure 6) and pathology (Figure 7), as well as other tissues (Supplementary Table S2). 

To assess viral burden as the infection progressed, the serum and tissue samples collected for cytokine profiling were also analyzed for viremia and viral loads. As described for the cytokine analyses, samples could only be obtained from a single day 6 animal. No infectious virus was detected in any of the tissues on day 2 p.i., but SFTSV was present consistently in the serum and all tissues except the intestines on day 4 and 6, suggesting that viremia and systemic dissemination likely started by day 3 p.i. The highest titers were observed in spleen, followed by liver and kidney. Viral replication was consistent with histopathology findings from the same tissue samples with documented inflammatory lesions, primarily in the spleen and liver on days 4 and 6 p.i., with two animals presenting with kidney lesions on day 4 (Figure 7). The splenic lesions consisted of acute, multifocal to coalescing, neutrophilic and histiocytic splenitis with variable lympholysis, lymphoid depletion, and vascular fibrinoid necrosis. The liver lesions consisted of acute, multifocal random, necrotizing, neutrophilic and histiocytic hepatitis. The renal lesions observed in the day 4 animals consisted of acute, multifocal to coalescing, tubular epithelial necrosis at the corticomedullary junction and deep cortex. 

### 2.3. Evaluation of FX06 and Favipiravir Combination Therapy for the Treatment of SFTSV Infection and Disease 

**Figure 8:**
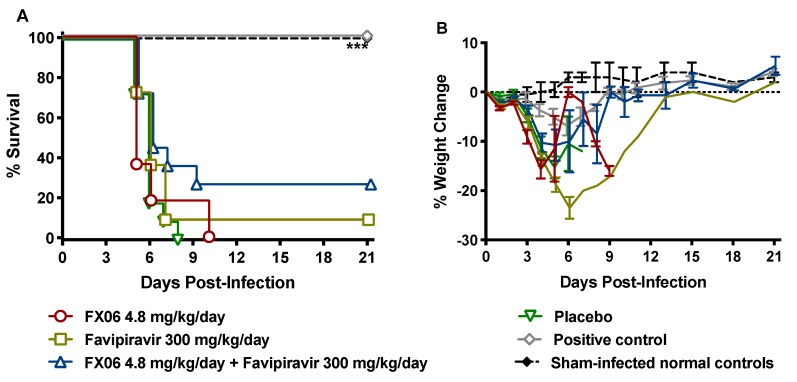
(**A**) Survival outcome and (**B**) percent weight change of IFNAR^-/-^ mice challenged with SFTSV and treated with FX06 alone or in combination with favipiravir. Mice in each group (n = 11) were infected SC with approximately 1 PFU of SFTSV and treated IP, twice daily for 7 days with 4.8 mg/kg/day FX06 beginning 3 days p.i., 300 mg/kg/day favipiravir beginning 5 days p.i., or both drugs in combination. A group of mice (n = 6) treated with 100 mg/kg/day of favipiravir starting 1 day p.i. was included as the positive control. Weight change is represented as the group mean and standard error of the percent change in weight of surviving animals relative to their starting weights on day 0. ***P < 0.001, compared to animals that received the placebos. Sham-infected normal controls are shown for comparison.

To evaluate the ability of FX06 to extend the therapeutic window of favipiravir through the control of vascular leak associated with SFTSV infection, we evaluated FX06 alone and in combination with favipiravir by assessing vascular leak, viral titers, and mortality in IFNAR^-/-^ mice challenged with 1 PFU of SFTSV. Groups of mice were treated by IP injection twice daily for 7 days with 4.8 mg/kg/day FX06 beginning on day 3 p.i., 300 mg/kg/day favipiravir beginning on day 5 p.i., both treatment regimens, or placebos (Supplementary Table S3). The drug combination of 4.8 mg/kg/day FX06 and 300 mg/kg/day favipiravir offered the greatest protection with three of 11 animals (27%) surviving the SFTSV challenge (Figure 8A). Only one out of 11 animals treated with favipiravir monotherapy started on day 5 p.i. survived the infection, and all 11 mice treated with FX06 alone succumbed by day 10 p.i. The positive control favipiravir treatment initiated one day after challenge performed as expected achieving 100% survival. Animal weights obtained during the course of the efficacy study are consistent with the survival data and indicate full recovery of the surviving animals (Figure 8B).

**Figure 9:**
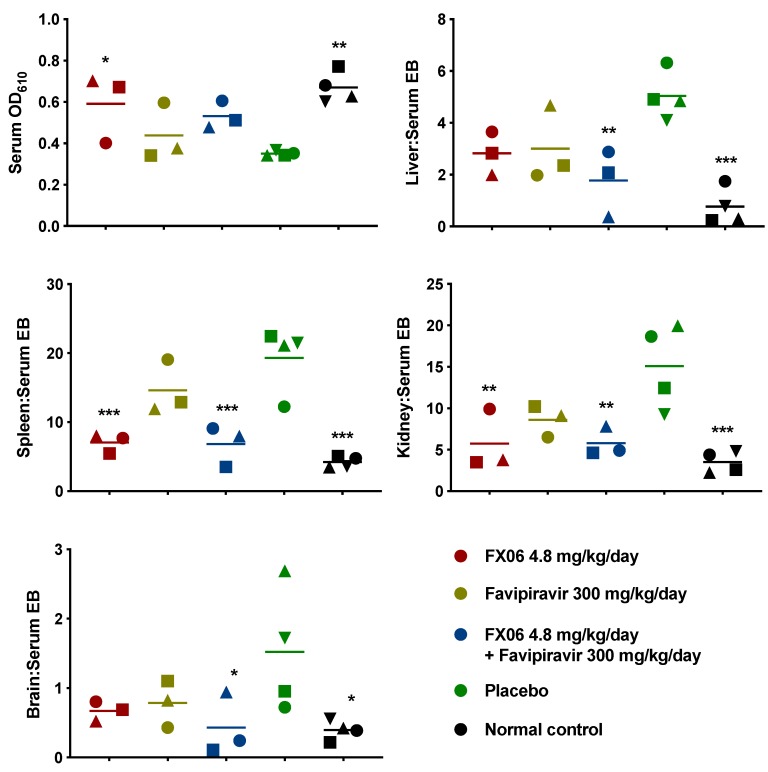
Evaluation of vascular permeability in mice sacrificed on day 5 p.i. of the initial FX06 and favipiravir combination therapy study. Serum data are reported as absorbance at 610 nm minus absorbance at 740 nm. Tissue data are reported as the mean tissue to serum ratio of absorbance/g tissue relative to serum absorbance values. Unique symbols in each sacrifice group represent values for the same animal across serum and all tissues. *** P < 0.001, ** P < 0.01, * P < 0.05, compared to animals that received the placebos.

Serum and tissue samples were collected from a subset of mice sacrificed on day 5 p.i. to assess the effect of the treatments on vascular leak and viral loads. Three animals each in the FX06, favipiravir, and combination treatment groups, and two animals in the placebo-treated group succumbed to infection just prior to sacrifice, reducing the number of animals available for analysis. As shown in Figure 9, the EBD tissue to serum ratio was significantly reduced in all of the tested tissues from mice treated with both FX06 alone or in combination with favipiravir, compared to animals treated with placebo. As expected, there was no observed reduction in serum or tissue viral titers in the mice treated with FX06 alone or in combination with favipiravir (Supplementary Figure S2). This is consistent with previous work in a mouse model of dengue shock syndrome [15]. In addition, it was not surprising that viral titers in the delayed favipiravir treatment group were not impacted, as this group of mice had only received a single treatment prior to collection of samples.

Having confirmed that FX06 treatment had the desired effect in reducing vascular leak, we conducted a second experiment to better resolve potential synergy between FX06 and favipiravir. Favipiravir treatment was initiated one day earlier (day 4 p.i.) at doses of 200 or 300 mg/kg/day alone or in combination with FX06 treatment, which began on day 3 p.i. (Supplementary Table S4). Our goal was to demonstrate that under conditions where favipiravir alone would only be partially protective (40–60% survival), combination therapy with FX06 would improve survival outcome. Unfortunately, our attempt to achieve approximately 50% protection with favipiravir monotherapy was unsuccessful as 87% and 93% protection was observed with the selected doses of 200 and 300 mg/kg/day, respectively, which did not differ significantly from the FX06 and favipiravir combination groups (Supplementary Figure S3). Consequently, there was little to no capacity to resolve beneficial interactions when the drugs were given in combination.

## 3. Discussion

With the expanding reach of SFTSV affecting densely populated areas of Asia [9], the need for effective therapeutics and vaccines has increased in urgency. To date there are three animal models of SFTSV infection where lethal disease is observed [11,12,13], but none have been characterized in terms of vascular leak, a cardinal feature of VHF believed to contribute to fatal outcome in severe cases of SFTS [1,16]. In the present study, we describe the natural history of disease and pathogenesis of SFTSV infection in IFNAR^-/-^ mice with a focus on profiling the inflammatory cytokine response and resolving the onset of vascular leak to facilitate comparisons between recent reports correlating cytokine levels in human cases of SFTS with disease severity [17,18,19,20,21]. Similar to several recent reports with other strains of SFTSV [22,23], we found that IFNAR^-/-^ mice are highly sensitive to the virus with a single PFU challenge dose achieving uniform lethality. Consequently, we selected a 3 PFU SC challenge dose in our natural history study to resemble a low-dose human exposure.

Our study is the first to investigate changes to vascular permeability during the course of experimental SFTSV infection. Our findings are indicative of vascular compromise starting on day 4 or 5 p.i., a time when elevated levels of prominent VHF proinflammatory cytokines such as IL-6, MCP-1, TNFα, IFNγ, RANTES and IL-1β were present in multiple animals in the serum, spleen and other tissues. Many of these cytokines are also markedly elevated and associated with severe cases of disease observed in human patients [17,18,19,20,21]. The correlation between human case studies and our data suggests that the IFNAR^-/-^ mouse SFTSV infection model may be useful for evaluating antiviral treatments combined with adjunctive therapy to limit vascular leak directly or through modulation of the intensity and duration of the proinflammatory cytokine response. Data obtained using the mouse model of SFTSV to investigate the use of agents that can mitigate vascular leak could be broadly applicable to other VHF viruses that cause similar clinical manifestations.

We envision that the optimal treatment strategy for SFTS and other VHFs would include potent direct-acting antiviral drugs that target the viral replication cycle and adjunctive therapy to control the deleterious effects of host-mediated vascular compromise. Through targeting of multiple factors such as viral polymerases, proteases or other essential viral enzymatic functions, the risk for development of drug resistance is mitigated. However, even the best drug cocktail may be ineffective in cases of advanced disease when the patient presents with vascular leak associated with hypercytokinemia. We hypothesized that by effectively reducing or reversing vascular leak, the therapeutic window for antiviral drug intervention could be extended. This approach was evaluated in the SFTSV IFNAR^-/-^ mouse model by combining favipiravir, previously shown to be efficacious in both mouse and hamster models of SFTS [12,24], with adjunctive FX06 therapy to treat the vascular disease component based on previous mouse data and human experience. 

FX06 is a 28-amino acid natural plasmin digest product of fibrin that has been tested in mouse models of dengue shock syndrome and LPS-induced lung inflammation, and was shown to significantly reduce edema and vascular leakage into the lung while improving survival outcome [15]. Because FX06 is not a direct-acting antiviral, the reduction in disease severity and improved survival outcome is attributed to reduced vascular permeability following FX06 administration. In addition, during the devastating 2014 Ebola virus disease outbreak in West Africa, FX06 was used as an experimental treatment for an exported case of Ebola-induced vascular leak syndrome [25]. The extravascular lung water index (EVLWI) used to quantitate pulmonary vascular leak during the course of the disease reached 25 mL/kg (normal range 3–7 mL/kg), prompting the initiation of FX06 treatment in an attempt to prevent shock and multiorgan failure. Administration of FX06 coincided with a substantial improvement of both vascular leak syndrome parameters and respiratory function [25]. However, with only a single successful outcome to support further consideration of FX06 therapy for the treatment of Ebola virus disease, additional supporting evidence demonstrating a beneficial outcome in animal models of VHF would support further investigation for human use.

In our initial experiment with the SFTSV IFNAR^-/-^ mouse infection model, we found that FX06 alone, and when combined with favipiravir, had the desired effect of reducing vascular leak. However, we only observed a subtle improvement in survival with the combination therapy compared to either drug alone, but the results did not achieve statistical significance. Our target survival range for the favipiravir monotherapy was 40%–60% based on previous work by Tani and colleagues [24]. In their study, they initiated 300 mg/kg/day favipiravir treatment on day 5 p.i. resulting in 50% survival when facing a 10^6^ TCID_50_ challenge dose of the SPL010 strain of SFTSV. Thus, it appears that the HB29 strain is more virulent in IFNAR^-/-^ mice, as challenge with 1 PFU and implementation of the same favipiravir treatment regimen starting 5 days p.i. resulted in a considerably lower survival rate of 9%. Because some of the mice began succumbing to infection as early as day 5 p.i., favipiravir therapy initiated on the same day was likely too late to achieve the desired survival rate of 40%–60%. In our attempt to address this issue in a follow-up experiment, we began favipiravir treatments one day earlier on day 4 p.i. Unfortunately, this resulted in near complete protection with the monotherapy alone, severely limiting our capacity to resolve a favorable interaction with the adjunctive FX06 therapy. In addition to the challenge of achieving partial protection, it is unclear whether vascular leak actually contributes to disease severity in IFNAR^-/-^ mice or is merely an end-stage manifestation. If the latter, then one would not expect FX06 to significantly impact survival outcome. This possibility and our study results highlight the complexity of conducting combination therapy experiments investigating the use of adjunctive host-directed therapies to supplement direct-acting antivirals.

## 4. Materials and Methods

### 4.1. Ethics Statement

All animal procedures used in this study complied with guidelines set by the USDA and Utah State University Institutional Animal Care and Use Committee protocol 10097. To minimize pain and distress, alternative endpoints for humane euthanasia included weight loss of 30% or greater or unresponsiveness.

### 4.2. Animals

Male and female, 4–9-week-old, IFN-alpha/beta receptor knockout (IFNAR^-/-^) mice (originally purchased from The Jackson Laboratory, MMRRC Stock No: 32045-JAX) were obtained from the breeding colony at Utah State University (Logan, UT), quarantined for a minimum of 5 days prior to challenge, and fed Harlan Lab Block and tap water ad libitum. 

### 4.3. Compounds

FX06 was kindly furnished by MChE GmbH (Vienna, Austria) and prepared in sterile saline. Favipiravir was generously provided by FUJIFILM Toyama Chemical Co., Ltd. (Toyama, Japan) and prepared in sterile water supplemented with 42.9 mg/mL meglumine excipient.

### 4.4. Virus

SFTSV, strain HB29, isolated in 2010 from a patient living in Hubei province of China, was obtained from Dr. Robert Tesh (World Reference Center for Emerging Viruses and Arboviruses, The University of Texas Medical Branch, Galveston, TX). The virus stock (5 × 10^6^ PFU/mL; 1 passage in Vero E6 cells) used was from a clarified cell culture lysate preparation. Virus stock was diluted in sterile medium and inoculated by SC injection of 0.2 mL containing the specified PFU of virus.

### 4.5. Determination of the SFTSV LD_90_ in IFNAR^-/-^ Mice

Male and female 4–5-week-old mice were sorted to minimize weight and sex differences across four challenge groups (n = 3 per group) and one sham-infected control group (n = 2). The challenge groups were inoculated SC with 0.2 mL containing log_10_ dilutions of 1000 to 1 PFU SFTSV. The animals were monitored daily for survival and body weight for 3 weeks. The LD_90_ was calculated using Prism (GraphPad).

### 4.6. Experimental Design to Characterize Vascular Leak and Hypercytokinemia

Male and female 5–week-old mice were weighed the day before infection with 3 PFU SFTSV (weight range of 15.5–22.3 g) and sorted to minimize weight and sex differences across all groups. Subsets of three to four mice were sacrificed on various days for analysis of vascular leak, cytokines, viral titers, and histopathology. The experiment was designed so that groups of animals designated for vascular leak analysis were sacrificed daily on days 2–6, and animals designated for cytokine analysis, viral titers, and histopathology were sacrificed on days 2, 4, and 6 (Supplementary Table S1). Sham-infected animals sacrificed on days 2, 4, and 6 were included as normal controls for all of the analyses.

### 4.7. Experimental Design of FX06 Combination Therapy with Favipiravir

For the initial study, male and female 6–9-week-old IFNAR^-/-^ mice were weighed the day before infection (weight range of 18.6–28.4 g) and sorted to minimize weight and sex differences across all groups. Animals in each group (n = 17) were infected with 1 PFU of SFTSV and treated twice per day (BID) for 7 days with either 4.8 mg/kg/day FX06 beginning on day 3 p.i. or 300 mg/kg/day favipiravir beginning on day 5 p.i. A third treatment group received both FX06 and favipiravir following the specified dosing regimen for each compound. A group of placebo-treated mice (n = 17) was included for comparison. Subsets of animals from each treatment group (n = 6) were selected prior to infection, treated in parallel, and sacrificed on day 5 for analysis of vascular leak and to obtain serum, liver, spleen, kidney, and brain tissue for analysis of viral titers. The remaining 11 animals were observed 21 days for morbidity and mortality. A group of six animals was treated with 100 mg/kg/day favipiravir, BID for 7 days, beginning 1-day p.i., as the positive control group for survival.

In a follow-up study, favipiravir treatment was initiated one day earlier (day 4 p.i.) at doses of 200 or 300 mg/kg/day. The mice (6–10-weeks of age) were weighed two days before infection and randomized to normalize weight and sex distribution across all treatment groups. This experiment was designed so that animals in each group (n = 15) were treated BID for 8 days with 4.8 mg/kg/day FX06 beginning on day 3 p.i. with 1 PFU of SFTSV, 200 mg/kg/day favipiravir beginning on day 4 p.i., or 300 mg/kg/day favipiravir beginning on day 4 p.i. Additional treatment groups included mice that received a combination of FX06 and one of the indicated favipiravir treatments. Also, a group of placebo-treated mice (n = 15) was included for comparison and a group of five animals was treated with 100 mg/kg/day favipiravir, BID for 7 days, beginning 1-day p.i., as the positive control group. All of the animals were observed 21 days for morbidity and mortality.

### 4.8. Vascular Permeability

Vascular permeability was assessed during the SFTSV infection as previously described [26]. Briefly, 200 μL of Evans blue dye (EBD) was injected retro-orbitally and whole blood was obtained 3 h later by submandibular bleed and processed for serum. Following euthanasia, animals were transcardially perfused with sterile phosphate buffered saline (PBS) prior to collection of tissue samples (liver, spleen, intestine, kidney, heart, lung and brain). Samples were incubated in formamide at 37 °C overnight, followed by brief centrifugation to pellet the tissue particulate, and the supernatant was evaluated by measuring absorbance at 610 nm and 740 nm. EBD content was reported as the optical density (OD) at 610 nm after subtraction of the OD reading at 740 nm to correct for hemoglobin content, and the tissue concentrations of EBD were normalized to the amount of dye present in the serum (1:10 diluted) to correct for animal-to-animal variation in the amount of EBD injected.

### 4.9. Cytokine Analysis

Whole blood was obtained by submandibular bleed and processed for serum. Following euthanasia and transcardial perfusion with PBS, tissue samples were homogenized in a fixed volume of minimum essential medium (MEM). Cytokine concentrations in the serum and tissue homogenates were analyzed using the Q-Plex Mouse Cytokine array (Quansys Biosciences, Logan, UT; complete list of cytokines on the panel are shown in Supplementary Table S2) and a mouse VEGF ELISA (ThermoFisher, USA). The assays were completed in accordance to the manufacturers’ protocols.

### 4.10. Virus Titer Determination

Virus titers were assayed using an infectious cell culture assay as previously described [27]. Following euthanasia and transcardial perfusion with PBS, tissue samples were homogenized in a fixed volume of MEM and the homogenates and serum were serially diluted and added to quadruplicate wells of Vero E6 (African green monkey kidney) cell monolayers in 96-well microtiter plates. Viral cytopathic effect (CPE) was determined 10 days after plating, and the 50% endpoints were calculated as described [28]. The lower limit of detection for serum samples was 1.67 log_10_ 50% cell culture infectious dose (CCID_50_) per ml and the lower limit of detection for tissues was in the range of 2.6–3.4 log_10_ CCID_50_/g. In samples presenting with undetectable virus, a value representative of the lower limit of detection was assigned for statistical analysis.

### 4.11. Histopathology

Tissue samples of the liver, spleen, intestine, kidney, heart, lung, and brain were preserved in 10% neutral-buffered formalin. Fixed tissue samples were processed and embedded in paraffin according to routine histologic techniques. Tissue sections, 5 µm thick, were stained with hematoxylin and eosin (H&E) and examined by light microscopy by board-certified pathologists who were blinded to the groups and day of sacrifice. Severity of tissue lesions was scored as follows: 0 = no lesions, 1 = minimal, 2 = mild, 3 = moderate, and 4 = severe.

### 4.12. Statistical Analysis

The Mantel-Cox log-rank test was used for analysis of Kaplan-Meier survival curves. A one-way analysis of variance (ANOVA) with Dunnett’s posttest to correct for multiple comparisons was used to compare differences in vascular permeability, cytokine concentrations, and viral titers. All statistical evaluations were done using Prism 8 (GraphPad Software, La Jolla, CA). 

## Supplementary Materials

The following are available online at https://www.mdpi.com/2076-0817/8/4/158/s1, Table S1: Study design to characterize SFTSV infection in IFNAR^-/-^ mice, Figure S1: Cytokine response in the serum (closed symbols) and spleen (open symbols) to SFTSV infection in IFNAR^-/-^ mice. Table S2: Cytokine response to SFTSV infection in IFNAR^-/-^ mice, Table S3: Study design to evaluate FX06 and favipiravir combination therapy to treat SFTSV infection and disease in IFNAR^-/-^ mice, Figure S2: Serum and tissue virus titers in IFNAR^-/-^ mice on day 5 p.i. of the initial FX06 and favipiravir combination therapy study, Table S4: Study design of the second evaluation of treatment with FX06 and favipiravir combination therapy to treat severe SFTSV infection and disease in IFNAR^-/-^ mice, Figure S3: Survival outcome of IFNAR^-/-^ mice in the follow-up study challenged with SFTSV and treated with FX06 alone or in combination with favipiravir.
