# Supplementary material for: Vascular Leak and Hypercytokinemia Associated with Severe Fever with Thrombocytopenia Syndrome Virus Infection in Mice
Source: Pathogens. 2019 Sep 21;8(4):158. doi: 10.3390/pathogens8040158 (PMC6963364; doi:10.3390/pathogens8040158)
Supplement: Supplementary file 1 [file pathogens-08-00158-s001.pdf]

Supplementary Table 1. Study design to characterize SFTSV infection in IFNAR$^{-/-}$ mice.

No./ Group	Group	Infected	Sacrifice Day	Analysis Performed
3	1	Y	2	Vascular leak
3	2	Y	2	Cytokines, viral titers, and histopathology
3	3	Y	3	Vascular leak
3	4	Y	4	Vascular leak
3	5	Y	4	Cytokines, viral titers, and histopathology
4	6	Y	5	Vascular leak
4	7	Y	6	Vascular leak
4	8	Y	6	Cytokines, viral titers, and histopathology
3	9	Sham	2,4,6	Vascular leak
3	10	Sham	2,4,6	Cytokines, viral titers, and histopathology

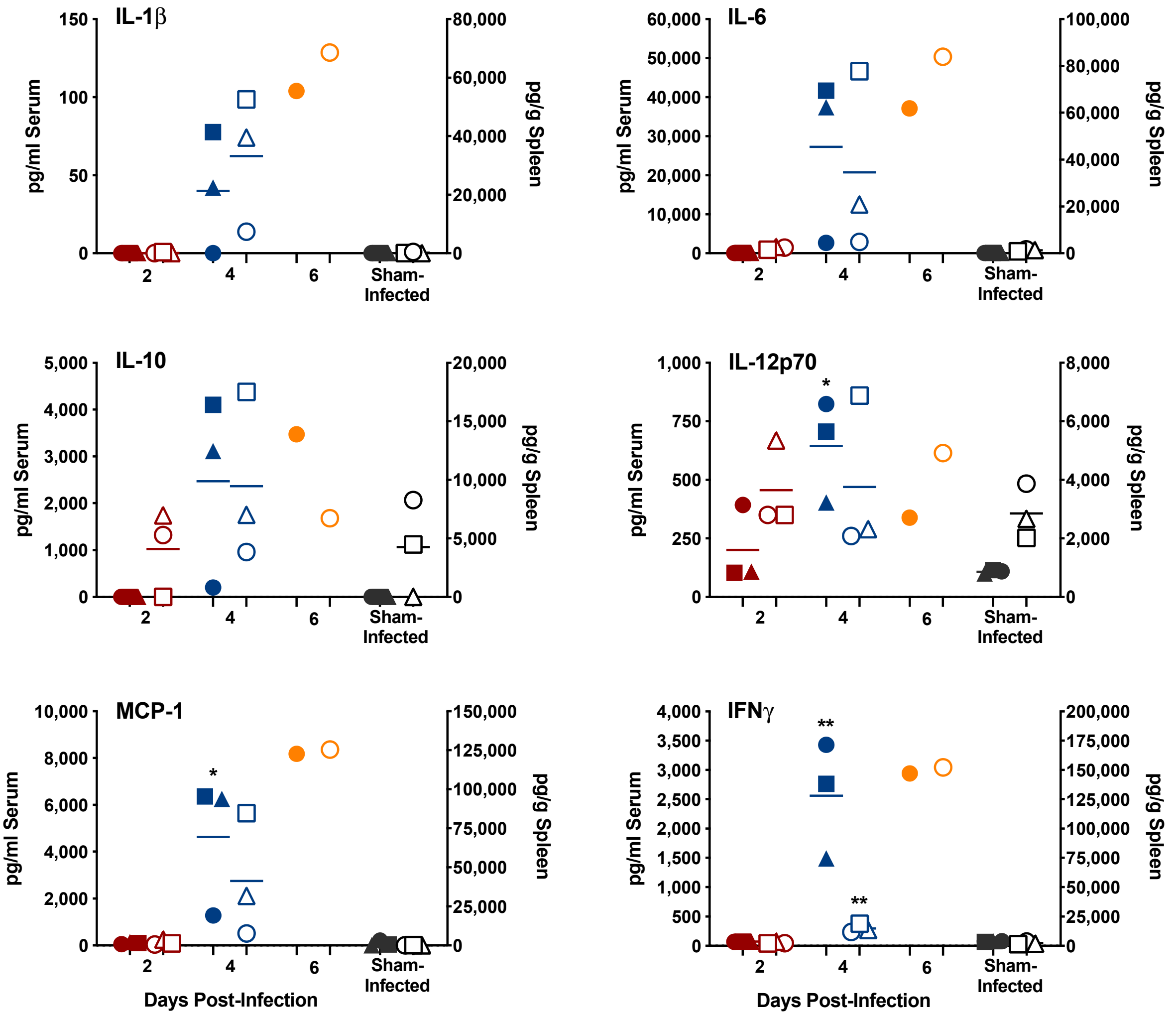

Supplementary Figure 1. Cytokine response in the serum (closed symbols) and spleen (open symbols) to SFTSV infection in IFNAR$^{-/-}$ mice. Each color represents the day p.i. that the animals were sacrificed. Unique symbols at each sacrifice day represent values for the same animals across all cytokines. Samples could not be obtained from three animals in the day-6 sacrifice group that succumbed to the infection prior to the designated time of sacrifice. ** $P < 0.01$, * $P < 0.05$ compared to sham-infected normal controls.

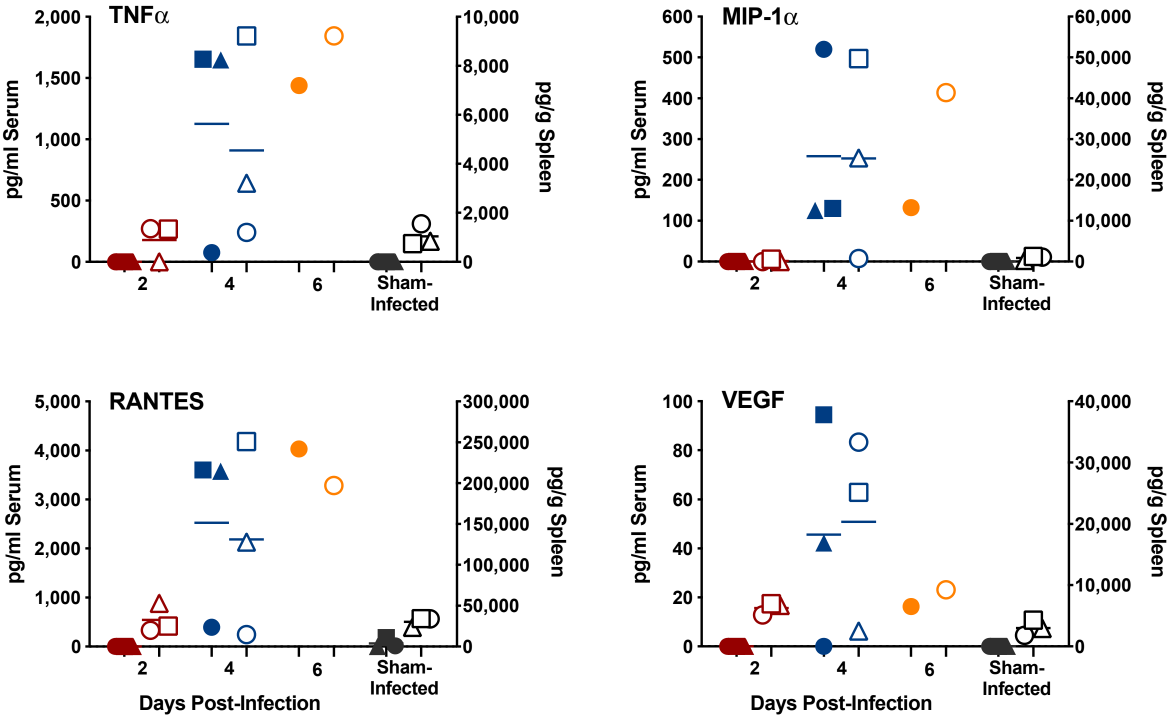

Supplementary Figure 1. Cytokine response in the serum (closed symbols) and spleen (open symbols) to SFTSV infection in IFNAR$^{-/-}$ mice. *(Continued).*

Supplementary Table 2. Cytokine response to SFTSV infection in IFNAR$^{-/-}$ mice.

Cytokine	Tissue	Day 2	Day 4	Day 6[c]	Control
**IL-1α**	Serum[a]	59.9 ± 26.2	122 ± 130	93.7	111 ± 59.3
	Liver[b]	1314 ± 389	61,717 ± 53,921	17,365	1264 ± 297
	Spleen	3062 ± 1493	593,624 ± 509,673	166,922	2572 ± 340
	Brain	237 ± 34.8	218 ± 34.3	1175	566 ± 535
	Intestine	741 ± 762	9625 ± 7209	6805	393 ± 250
	Kidney	1420 ± 279	8183 ± 6462	2089	1810 ± 1115
	Heart	1098 ± 797	2041 ± 1458	882	598 ± 188
	Lung	917 ± 309	1073 ± 206*	992	402 ± 79.5
**IL-1β**	Serum	0 ± 0.0	39.9 ± 38.9	104	0 ± 0.0
	Liver	1625 ± 468	5176 ± 3584	31,800	1784 ± 646
	Spleen	130 ± 225	33,134 ± 23,239	68,596	144 ± 249
	Brain	77.2 ± 58.3	2.6 ± 4.4	2941	67.8 ± 59.0
	Intestine	744 ± 825	9792 ± 9065	10,387	525 ± 144
	Kidney	2393 ± 614	2842 ± 1021	4361	2802 ± 1558
	Heart	1015 ± 737	0 ± 0.0	3008	192 ± 132
	Lung	386 ± 269	301 ± 337	3152	317 ± 59.2
**IL-2**	Serum	29.0 ± 12.5	40.7 ± 22.3	62.2	43.6 ± 8.4
	Liver	1531 ± 434	2706 ± 1522	1432	1564 ± 457
	Spleen	1277 ± 645	1731 ± 1664	1227	1155 ± 497
	Brain	274 ± 74.6	204 ± 19.3*	657	388 ± 59.1
	Intestine	430 ± 164	581 ± 492	121	277 ± 24.2
	Kidney	2338 ± 576	2008 ± 552	1470	2826 ± 1688
	Heart	1765 ± 1250	1121 ± 243	617	1008 ± 310
	Lung	1152 ± 741	667 ± 335	713	508 ± 11.4
**IL-3**	Serum	25.9 ± 2.8	25.1 ± 1.5	25.4	26.6 ± 0.8
	Liver	651 ± 180	1380 ± 839	686	682 ± 193
	Spleen	939 ± 277	1127 ± 841	948	680 ± 158
	Brain	166 ± 25.6	144 ± 21.0*	381	211 ± 18.7
	Intestine	362 ± 117	375 ± 163	108	204 ± 17.5
	Kidney	1103 ± 238	879 ± 317	659	1234 ± 747
	Heart	1034 ± 566	495 ± 89.2	388	565 ± 166
	Lung	628 ± 263	433 ± 165	471	276 ± 43.5

Supplementary Table 2. Cytokine response to SFTSV infection in IFNAR$^{-/-}$ mice. *(Continued)*

Cytokine	Tissue	Day 2	Day 4	Day 6[c]	Control
**IL-4**	Serum[a]	68.6 ± 2.3*	71.8 ± 1.7	79.1	74.5 ± 2.3
	Liver[b]	1053 ± 305	1827 ± 1039	1076	1098 ± 316
	Spleen	2479 ± 929	2078 ± 1611	1699	1937 ± 608
	Brain	439 ± 15.8	307 ± 28.9	967	494 ± 84.0
	Intestine	868 ± 296	759 ± 235	274	513 ± 43.4
	Kidney	1631 ± 319	1361 ± 345	1042	1752 ± 826
	Heart	2318 ± 1520	1333 ± 295	1004	1365 ± 470
	Lung	1597 ± 699	1095 ± 330	1044	672 ± 107
**IL-5**	Serum	236 ± 81.6	344 ± 183	671	220 ± 33.5
	Liver	6550 ± 1799	11,035 ± 5985	6858	6980 ± 2264
	Spleen	7452 ± 2972	8210 ± 7066	7936	7242 ± 1861
	Brain	1270 ± 140**	1318 ± 273**	3889	2105 ± 60.7
	Intestine	2578 ± 1010	3762 ± 1947	843	2133 ± 1061
	Kidney	9380 ± 1996	8129 ± 2127	6854	10,986 ± 4993
	Heart	8135 ± 3911	4034 ± 1396	3628	5024 ± 1123
	Lung	5239 ± 1665	4309 ± 1739	7283	2676 ± 808
**IL-6**	Serum	52.0 ± 2.9	27,257 ± 21,405	37,115	59.2 ± 13.9
	Liver	3253 ± 1071	8657 ± 4330	22,681	2581 ± 747
	Spleen	2233 ± 579	34,504 ± 38,308	83,998	1308 ± 410
	Brain	438 ± 166	625 ± 139	3056	594 ± 127
	Intestine	825 ± 361	3199 ± 3497	186	443 ± 112
	Kidney	3822 ± 1006	42,268 ± 36,043	24,040	5325 ± 3711
	Heart	3299 ± 2131	5810 ± 4695	5823	1278 ± 578
	Lung	1253 ± 103	1805 ± 555*	4347	835 ± 166
**IL-10**	Serum	0 ± 0.0	2472 ± 2027	3471	0 ± 0.0
	Liver	2703 ± 688	4012 ± 1955	2286	2475 ± 511
	Spleen	4087 ± 3638	9455 ± 7148	6730	4258 ± 4144
	Brain	653 ± 743	1095 ± 142	3781	1689 ± 324
	Intestine	605 ± 1047	1069 ± 1411	340	0 ± 0.0
	Kidney	3960 ± 687	3972 ± 1011	2841	4485 ± 1565
	Heart	7164 ± 5144	4956 ± 1884	4321	4979 ± 1855
	Lung	5096 ± 2036	3865 ± 1489	3436	1994 ± 229

Supplementary Table 2. Cytokine response to SFTSV infection in IFNAR$^{-/-}$ mice. *(Continued)*

Cytokine	Tissue	Day 2	Day 4	Day 6[c]	Control
**IL-12p70**	Serum[a]	202 ± 166	644 ± 217*	339	108.1 ± 7.4
	Liver[b]	3190 ± 1770	4808 ± 2701	2623	2771 ± 722
	Spleen	3649 ± 1466	3759 ± 2704	4920	2852 ± 937
	Brain	682 ± 71.8	653 ± 111	7858	802 ± 209
	Intestine	1377 ± 538	1570 ± 944	405	1544 ± 949
	Kidney	4093 ± 862	3672 ± 1533	2574	4806 ± 2388
	Heart	4209 ± 2576	2282 ± 662	1601	2326 ± 499
	Lung	3205 ± 1117	1758 ± 720	2562	1290 ± 61.8
**IL-17**	Serum	26.5 ± 0.0	79.0 ± 47.8	123	32.7 ± 6.7
	Liver	1258 ± 477	2660 ± 1613	1678	1340 ± 534
	Spleen	1703 ± 596	2041 ± 2106	2317	913 ± 422
	Brain	258 ± 28.8	260.0 ± 52.4	1706	281 ± 92.0
	Intestine	876 ± 681	693 ± 380	160	307 ± 55.4
	Kidney	2184 ± 562	2002 ± 650	1797	2421 ± 1347
	Heart	1557 ± 967	638 ± 246	1243	817 ± 218
	Lung	955 ± 644	678 ± 252	1537	422 ± 130
**MCP-1**	Serum	98.9 ± 40.5	4632 ± 2907*	8176	79.6 ± 109
	Liver	770 ± 196	27,376 ± 21,744	42495	877 ± 218
	Spleen	1832 ± 1654	41,358 ± 39,408	125,470	44.8 ± 77.7
	Brain	254 ± 221	2126 ± 1185	68,876	403 ± 21.9
	Intestine	388 ± 370	12,715 ± 18,140	1345	49.0 ± 84.9
	Kidney	1049 ± 190	18,757 ± 13,908	37,505	1359 ± 667
	Heart	894 ± 559	17,783 ± 13,902	52,189	805 ± 370
	Lung	1482 ± 1357	15,378 ± 3625***	62,572	375 ± 130
**IFNγ**	Serum	71.1 ± 2.3	2561 ± 984**	2941	74.8 ± 5.6
	Liver	2238 ± 866	4598 ± 2518	12,285	2229 ± 744
	Spleen	2608 ± 841	14,715 ± 3706**	152,313	2333 ± 1169
	Brain	479 ± 19.5**	433 ± 51.7***	2414	724.2 ± 59.8
	Intestine	1002 ± 307	1299 ± 667	4313	575 ± 163
	Kidney	3834 ± 898	5505 ± 2454	7064	4875 ± 3638
	Heart	3527 ± 2675	1566 ± 171	2065	1777 ± 686
	Lung	1547 ± 511	1418 ± 680	3099	954 ± 135

Supplementary Table 2. Cytokine response to SFTSV infection in IFNAR$^{-/-}$ mice. *(Continued)*

Cytokine	Tissue	Day 2	Day 4	Day 6[c]	Control
**TNFα**	Serum[a]	0 ± 0.0	1126 ± 910	1439	0 ± 0.0
	Liver[b]	716 ± 206	1443 ± 848	1050	826 ± 5.0
	Spleen	895 ± 775	4545 ± 4169	9222	1047 ± 439
	Brain	251 ± 138	214.8 ± 28.7	1060	182 ± 123
	Intestine	353 ± 331	680 ± 313	98.2	27.0 ± 46.8
	Kidney	909 ± 193	1405 ± 530	913	1261 ± 622
	Heart	1061 ± 991	815 ± 380	719	746 ± 572
	Lung	141 ± 99*	497 ± 86	766	412 ± 128
**MIP-1α**	Serum	0 ± 0.0	258 ± 226	133	0 ± 0.0
	Liver	1429 ± 597	4728 ± 3675	2287	1491 ± 445
	Spleen	191 ± 330	25,295 ± 24,466	41,380	915 ± 495
	Brain	140 ± 243	238 ± 66.4	1109	230 ± 205
	Intestine	3405 ± 5719	1066 ± 114	1609	93.7 ± 162
	Kidney	2321 ± 1335	3051 ± 1787	2437	2170 ± 1107
	Heart	1494 ± 1492	1442 ± 980	897	669 ± 145
	Lung	1322 ± 1670	1754 ± 1524	1146	269 ± 15.2
**GM-CSF**	Serum	0 ± 0.0	13.2 ± 15.4	45.4	1.0 ± 1.8
	Liver	865 ± 264	1693 ± 931	1020	1025 ± 394
	Spleen	294 ± 444	1292 ± 1296	2115	206 ± 182
	Brain	117 ± 112	56.3 ± 57.4	664	219 ± 52.2
	Intestine	173 ± 150	476 ± 266	125	102.5 ± 83.6
	Kidney	1434 ± 433	1157 ± 305	854	1582 ± 616
	Heart	1112 ± 888	576 ± 198	366	388 ± 183
	Lung	433 ± 493	384 ± 315	291	277 ± 94.4
**RANTES**	Serum	0 ± 0.0	2524 ± 1846	4032	64.3 ± 99.9
	Liver	1525 ± 666	10,534 ± 9811	2339	1870 ± 1058
	Spleen	32,700 ± 17,685	131,034 ± 118,139	197,114	30,451 ± 6155
	Brain	0 ± 0.0	162 ± 280	892	93.6 ± 162
	Intestine	7994 ± 7786	10,823 ± 11,486	388	66.1 ± 114
	Kidney	1910 ± 832	13,304 ± 10,328	5940	3425 ± 2944
	Heart	1638 ± 2837	4732 ± 4878	1694	1604 ± 2779
	Lung	1039 ± 1799	8726 ± 9992	4332	1002 ± 1358

Supplementary Table 2. Cytokine response to SFTSV infection in IFNAR$^{-/-}$ mice. *(Continued)*

Cytokine	Tissue	Day 2	Day 4	Day 6[c]	Control
**VEGF**	Serum[a]	0 ± 0.0	45.6 ± 47.4	16.3	0 ± 0.0
	Liver[b]	1.7E+06 ± 1.4E+06	5.1E+06 ± 3.5E+06	9.0E+07	1.8E+07 ± 2.6E+07
	Spleen	6268 ± 978	20,335 ± 15,971	9242	3008 ± 1243
	Brain	895 ± 701	404.8 ± 64.9	1671	1064 ± 594
	Intestine	60,561 ± 76,204	541,530 ± 189,819**	138,345	43,568 ± 37,863
	Kidney	9.9E+05 ± 1.5E+06	2.2E+06 ± 1.9E+06	2.90E+05	7.8E+06 ± 1.0E+07
	Heart	35,060 ± 21,095	29,528 ± 6482	60,114	18,052 ± 12,628
	Lung	78,180 ± 41,856	86,955 ± 122,938	15,1642	42,312 ± 16,170

[a] All serum values expressed as pg/mL serum.
[b] All tissue values reported as pg/g tissue.
[c] Three of the four animals succumbed to the infection prior to the time of sacrifice.
*** $P < 0.001$, ** $P < 0.01$, * $P < 0.05$ compared to normal controls (n = 3).

Supplementary Table 3. Study design to evaluate FX06 and favipiravir combination therapy to treat SFTSV infection and disease in IFNAR$^{-/-}$ mice.

No./ Group	Group	Infected	Compound & Dose	Treatment Schedule	Observations & Testing
11	1	Y	FX06 4.8 mg/kg/day	Beginning 3 d p.i., BID x 7 days	Observed for weight loss and mortality through day 21
			Placebo	Beginning 5 d p.i., BID x 7 days	
11	2	Y	Placebo	Beginning 3 d p.i., BID x 7 days	
			Favipiravir 300 mg/kg/day	Beginning 5 d p.i., BID x 7 days	
11	3	Y	FX06 4.8 mg/kg/day	Beginning 3 d p.i., BID x 7 days	
			Favipiravir 300 mg/kg/day	Beginning 5 d p.i., BID x 7 days	
11	4	Y	Placebo	Beginning 3 d p.i., BID x 7 days	
			Placebo	Beginning 5 d p.i., BID x 7 days	
6	5	Y	Favipiravir 100 mg/kg/day	Beginning 1 d p.i., BID x 7 days	
2	6	Sham	Sham-infected controls for body weight		
6	1	Y	FX06 4.8 mg/kg/day	Beginning 3 d p.i., BID until sacrifice	Sacrificed on day 5 p.i. for vascular leak and viral titer analyses
			Placebo	Beginning 5 d p.i., BID until sacrifice	
6	2	Y	Placebo	Beginning 3 d p.i., BID until sacrifice	
			Favipiravir 300 mg/kg/day	Beginning 5 d p.i., BID until sacrifice	
6	3	Y	FX06 4.8 mg/kg/day	Beginning 3 d p.i., BID until sacrifice	
			Favipiravir 300 mg/kg/day	Beginning 5 d p.i., BID until sacrifice	
6	4	Y	Placebo	Beginning 3 d p.i., BID until sacrifice	
			Placebo	Beginning 5 d p.i., BID until sacrifice	
4	6	Sham	Sham-infected controls for vascular leak and viral titers		

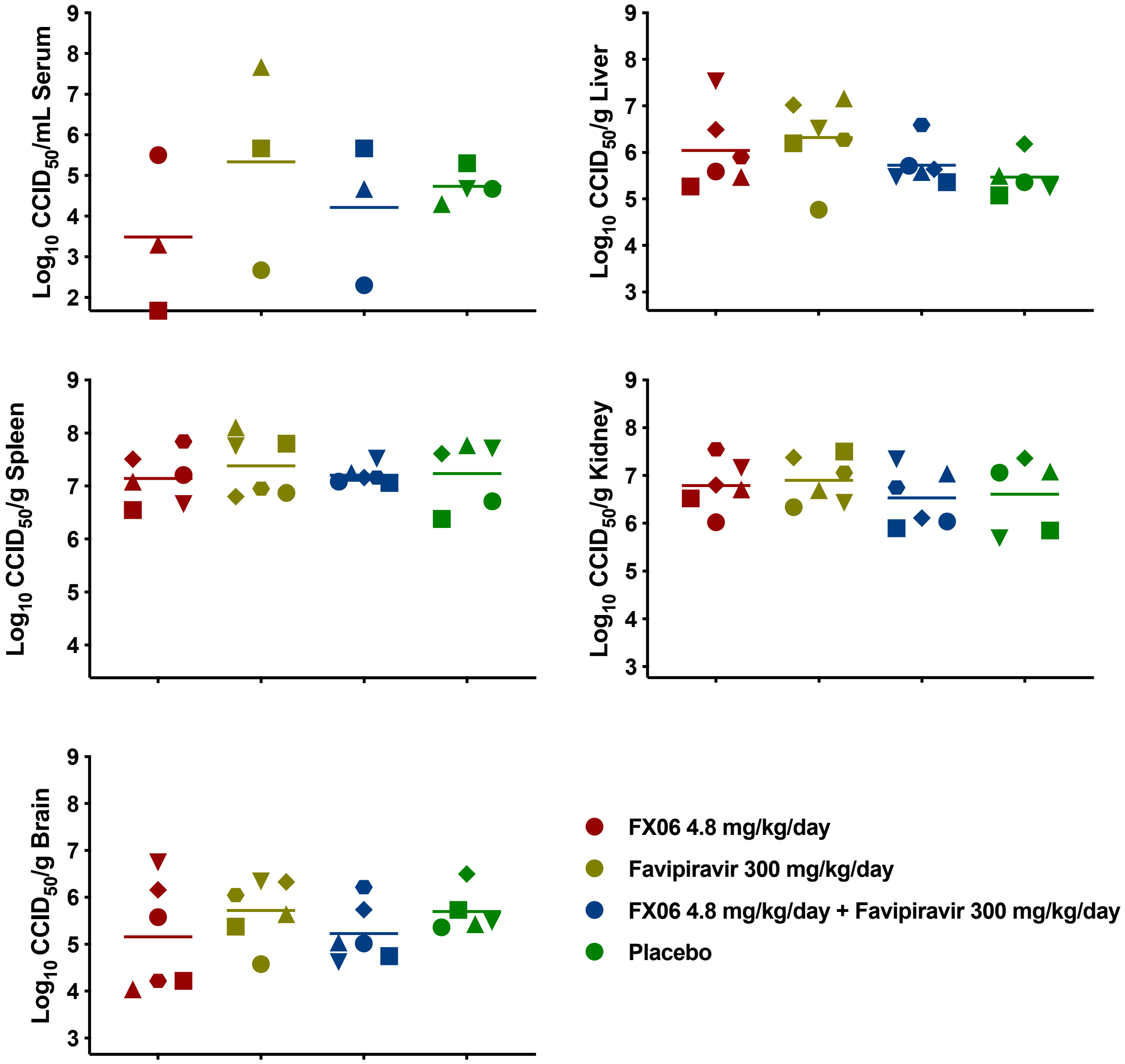

Supplementary Figure 2. Serum and tissue virus titers in IFNAR$^{-/-}$ mice on day 5 p.i. of the initial FX06 and favipiravir combination therapy study. Groups of animals were sacrificed on day 5 p.i. for analysis of serum, liver, spleen, kidney, and brain virus titers. The x-axes represent the respective lower limits of detection for serum and the indicated tissues. Unique symbols in each treatment group correspond to the same animal across the serum and all tissues.

Supplementary Table 4. Study design of the second evaluation of treatment with FX06 and favipiravir combination therapy to treat severe SFTSV infection and disease in IFNAR$^{-/-}$ mice.

No./ Group	Group	Infected	Compound & Dose	Treatment Schedule	Observations & Testing
15	1	Y	FX06 4.8 mg/kg/day	Beginning 3 d p.i., BID x 8 days	Observed for weight loss and mortality through day 21
			Placebo	Beginning 4 d p.i., BID x 8 days	
15	2	Y	FX06 4.8 mg/kg/day	Beginning 3 d p.i., BID x 8 days	
			Favipiravir 200 mg/kg/day	Beginning 4 d p.i., BID x 8 days	
15	3	Y	FX06 4.8 mg/kg/day	Beginning 3 d p.i., BID x 8 days	
			Favipiravir 300 mg/kg/day	Beginning 4 d p.i., BID x 8 days	
15	4	Y	Placebo	Beginning 3 d p.i., BID x 8 days	
			Placebo	Beginning 4 d p.i., BID x 8 days	
15	5	Y	Placebo	Beginning 3 d p.i., BID x 8 days	
			Favipiravir 200 mg/kg/day	Beginning 4 d p.i., BID x 8 days	
15	6	Y	Placebo	Beginning 3 d p.i., BID x 8 days	
			Favipiravir 300 mg/kg/day	Beginning 4 d p.i., BID x 8 days	
5	7	Y	Favipiravir 100 mg/kg/day	Beginning 1 d p.i., BID x 7 days	
3	8	Sham	Sham-infected controls for body weight		

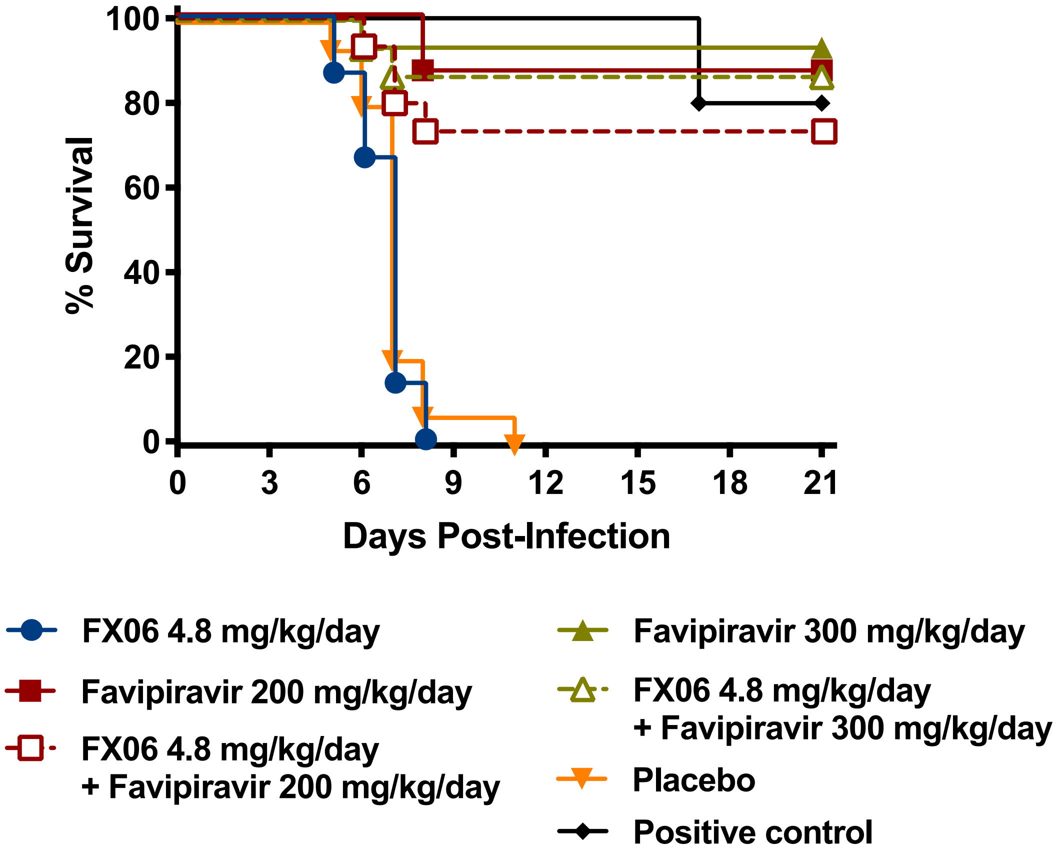

Supplementary Figure 3. Survival outcome of IFNAR$^{-/-}$ mice in the follow-up study challenged with SFTSV and treated with FX06 alone or in combination with favipiravir. Mice in each group (n = 15) were infected SC with approximately 1 PFU of SFTSV and treated IP, twice daily for 8 days with 4.8 mg/kg/day FX06 beginning 3 days p.i., 200 mg/kg/day favipiravir or 300 mg/kg/day favipiravir beginning 4 days p.i., or FX06 in combination with either dose of favipiravir. A group of mice (n = 5) treated with 100 mg/kg/day of favipiravir starting 1 day p.i. was included as the positive control.
